# Ultrasound-guided phrenic nerve block for intraoperative persistent hiccups: a case report

**DOI:** 10.1186/s12871-018-0589-2

**Published:** 2018-09-05

**Authors:** Yong Zhang, Fuhong Duan, Wuhua Ma

**Affiliations:** 1 Department of Anesthesiology, The First Affiliated Hospital of Guangzhou University of Chinese Medicine, 16# Jichang Road, Guangzhou, 510405 China; 2 Department of MRI, The First Affiliated Hospital of Zhengzhou University, No. 1, Jianshe East Road, Zhengzhou, 450052 China

**Keywords:** Hiccup, Ultrasound-guided, Nerve block

## Abstract

**Background:**

Continuous hiccups during surgery not only affect the surgical procedure, they can also cause adverse effects for the patient. Apart from active investigation of the cause of the hiccups, their timely termination is also necessary.

**Case presentation:**

We reported a case of a 70-year-old woman with continuous intraoperative hiccups that appeared during vaginal hysterectomy under low continuous epidural anesthesia. After the ineffectiveness CO_2_ repeated inhalation and intravenous administration of chlorpromazine and methoxychlorpromide, we performed unilateral phrenic nerve block under ultrasound guidance. Hiccups were terminated without any related complications.

**Conclusions:**

During intraoperative continuous hiccups, ultrasound guided phrenic nerve block may be a suitable treatment option when physical methods and drug therapy are not effective. However, given the absence of a vital risk related to hiccups, this block should imply the complete absence of any respiratory contraindication and a prolonged postoperative respiratory monitoring.

## Background

Hiccups are an unpleasant experience; they usually last for a short duration of time and do not cause any harm. However, long-term hiccupping can cause serious complications such as dehydration, weight loss, fatigue, insomnia, psychosis, depression, arrhythmia [1]. Persistent hiccups rarely occur during an ongoing operation. However, when they do, the hiccups affect not only the patient’s respiratory and circulatory function, they also interfere with the surgical procedure. Under these circumstances, it is necessary to interrupt the hiccups as soon as possible. A phrenic nerve block may be a suitable treatment choice because it can quickly and effectively terminate hiccups when physical or medical treatments are not effective. An ultrasound-guided block can improve the success rate and reduce the potential for complications [2]. The effects of surgery and anesthesia on the patient’s respiratory system should be highly considered when performing an intraoperative phrenic nerve block. Here, we report a case of an intraoperative persistent hiccups that appeared during vaginal hysterectomy performed under low continuous epidural anesthesia. We administered a unilateral phrenic nerve block under ultrasound guidance, and the hiccups were terminated successfully without any related complications.

## Case presentation

**Fig. 1:**
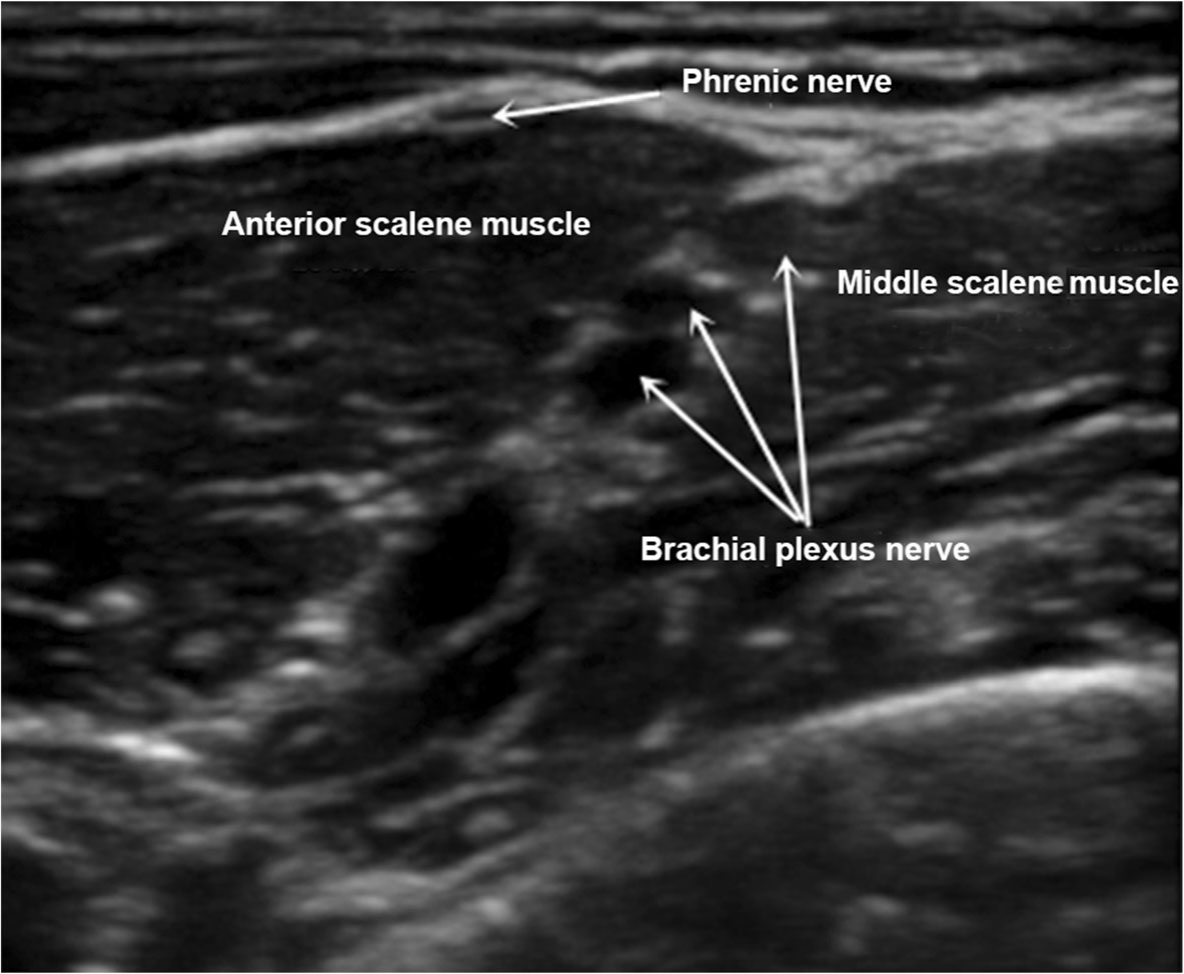
The phrenic nerve ultrasonography of the right neck

The patient involved has consented to the publication of her case and signed the consent form. A 70-year-old woman was hospitalized for third degree uterine prolapse in the First Affiliated Hospital of Guangzhou University of Chinese Medicine on February 10, 2016. Vaginal hysterectomy under continuous epidural anesthesia was indicated. She was rated class I risk using the American Society of Anesthesiologists (ASA) criteria and did not have any cardiopulmonary dysfunction or gastrointestinal disease. The preoperative blood tests were normal. The arterial blood pressure (ABP) was 145/85 mmHg. The peripheral oxygen saturation (SpO2) measured using pulse oximetry was 98%. The heart rate (HR) was 70 beats per minute. The results of the blood gas analysis, performed after the patient entered the operating room, were as follows: arterial partial pressure of oxygen (PaO2), 90 mmHg; and arterial partial pressure of carbon dioxide (PaCO2), 39 mmHg. The patient was placed in the left lateral position, and a continuous epidural anesthesia was performed in the L2 – L3 space. After successful puncture, a catheter was inserted in cephalad direction 4 cm beyond the tip of the needle. The patient was then placed in a supine position and given oxygen continuously via nasal catheter with an oxygen flow rate of 2 L/minute. A test dose of 3 mL 1.5% lidocaine was administered through the epidural catheter, followed by 8 ml of a 1.5% lidocaine-0.5% bupivacaine mixture. Sensory block was shown to be complete within 10 min, and the upper level of the block was at the level of T8. At this time, ABP was 130/70 mmHg, SPO2 was 99%, and HR was 82 beats per minute. A continuous intravenous infusion of dexmedetomidine 0.3 μg/kg/h was administered. The patient developed hiccups approximately 30 min after the start of surgery, about 10 times per minute at first, gradually increasing to 30 times per minute, 10 min later. At this time, ABP was 150/90 mmHg, SPO2 was 98%, and HR was 90 beats per minute. The persistent hiccups interrupted the operation. In order to terminate the hiccups, we first used repeated CO2 inhalation for 5 min, followed by intravenous injection of 25 mg chlorpromazine and 20 mg metoclopramide. There was no effect after 20 min. The abnormal movement of the diaphragm was confirmed using an ultrasound examination. It showed a spastic contraction of the patient’s right diaphragm. We decided to block the right phrenic nerve under the guidance of a high resolution portable ultrasound unit (Italy, Yum Mylab One). With the patient’s head towards the left, the ultrasound probe (frequency, 8 MHz) was placed on the right side of the neck. The axial scanning of the neck along the surface of the anterior scalene muscle, showed that the phrenic nerve rounded the anterior scalene muscle from the outside to the inside, and coursed through the trench between the common carotid artery and anterior scalene muscle (Fig. 1). Using an in-plane technique, when the needle was close to the phrenic nerve, 5 ml 0.4% ropivacaine was injected around the phrenic nerve. The hiccups gradually stopped after approximately 5 min. At this time, ABP was 135/75 mmHg, SPO2 was 99%, HR was 72 beats per minute, PaO2 was 88 mmHg, and PaCO2 was 41 mmHg. The patient reported no discomfort, and no related complications were observed. The operation resumed and ended 80 min later. The patient was kept in the intensive care unit for 24 h and was discharged 7 days after the surgery. No hiccups or phrenic nerve block-related complications were observed after the operation. No related complications were reported on the telephonic follow-up one month after being discharged.

## Discussion and conclusions

A hiccup is an involuntary spasmodic contraction of the diaphragm, which is an unconscious spinal reflex and has a complete reflex arc. The afferent nerves are the phrenic nerve, vagus nerve, and sympathetic nerve (T6 – T12), and the reflex center consists of the brainstem, phrenic nucleus, medullary reticular formation, and hypothalamus. The efferent nerves are the phrenic nerve and intercostal nerves, and the effectors comprise of the diaphragm, intercostal muscle, and anterior scalene muscle [3]. There are various causes of hiccups, and the most common are diseases located in the central nervous system and peripheral nervous pathways and digestive system diseases [4].

In the current patient, who had continuous hiccups during surgery, the cause may be related to uterine stretching during gynecological surgery, which caused stretching of other organs and tissues in the abdominal cavity and stimulation of the phrenic nerve leading to causing spasm of the diaphragm. In addition, hiccups may occur when, as a result of anxiety, a patient swallows a lot of cold air into the stomach before surgery [5]. It has been reported that bupivacaine epidural anesthesia could also induce hiccups [6].

Continuous hiccups during surgery is a rare phenomenon; however, it can severely influence both the patient and the surgical procedure. Stuth et al. [7] reported a case where continuous hiccups induced pulmonary edema, and Rullo et al. [8] reported a case in which continuous hiccups caused left common iliac artery perforation. Therefore, timely termination of continuous hiccups during surgery is essential.

Various methods can be used for treating hiccups [6], including physical treatment (rebreathing carbon dioxide), drug treatment (chlorpromazine), and nerve block. When physical and pharmacological treatments are not successful, a phrenic nerve block may be a good option; it can quickly block the hiccup reflex pathway and terminate the hiccups. As the phrenic nerve is thin, blind puncture has a higher failure rate, and may easily damage the nerve, blood vessels, and peripheral tissues. A high-resolution ultrasound scan can accurately distinguish the phrenic nerve and its surrounding anatomy. As a result, phrenic nerve block under ultrasound guidance has an improved success rate and can also decrease the damage to nearby nerves and blood vessels [2]. Furthermore, it has been reported that five cancer patients with intractable hiccups underwent unilateral phrenic nerve block under ultrasound guidance and all patients had satisfactory results without any complications [9].

While phrenic nerve block can effectively treat hiccups, it may also affect respiratory function [10]. Renes et al. [11] performed a phrenic nerve block for a patient with intractable hiccups under ultrasound guidance and subsequently found that the three parameters of FEV1, FVC, and PEF decreased by 12%, 13%, and 12%, respectively. A phrenic nerve block can highly impact the respiratory function; however, for patients with normal respiratory function, it should not cause adverse clinical consequences [3]. Wilkins et al. [12] suggested that for patients without respiratory insufficiency, paralysis of one side of the diaphragm can be compensated by the other side of the diaphragm and intercostal muscles, the SPO2 will not decrease, and patients will not develop dyspnea.

In this case, the patient was a 70-year-old woman, so we monitored the invasive arterial blood pressure and blood gas analysis. The patient was administered a low segmental epidural anesthesia with anesthesia reaching the level of T8 without involving the respiratory muscles, and the patient’s respiratory function was not affected. Therefore, it seemed safe to perform the unilateral phrenic nerve block using ultrasound guidance. Considering the long operation condition and to avoid recurring hiccup, we chose a long-acting local anesthetic with less cardiotoxicity, ropivacaine. Kang et al. [2] used ropivacaine to effectively block the phrenic nerve conduction and stop hiccups. In this case, the patient’s hiccups stopped immediately after the injection of ropivacaine and there was no desaturation or symptoms of dyspnea. However, when preoperative respiratory insufficiency or using a high segmental epidural anesthesia, as a result of inhibition of the patient’s respiratory function, the risks of dyspnea should be taken into account when administering a phrenic nerve block.

Several limitations of the current case report should be considered. First, we did not report information on the recovery of phrenic nerve function and the timing of hemi-diaphragm movement. Second, we did not observe the spread of local anesthetics and its effect on brachial plexus under ultrasound. In addition, we did not pay attention to the effect of phrenic nerve block on other organ functions.

Continuous hiccup during surgery is a rare occurrence that can lead to severe outcomes. Hence, when choosing the methods to treat hiccups, we should consider the causes of hiccups, the surgery, and the anesthesia. This article described only one case, which was successfully treated with phrenic nerve block, a technique which may not be suitable for all cases with hiccups during surgery. Nevertheless, this case may provide relevant evidence for selecting suitable treatment strategies for treating continuous hiccups that develop during surgery.

## Abbreviations

ABP: Arterial blood pressure
HR: Heart rate
PaCO2: Partial pressure of carbon dioxide
PaO2: Partial pressure of oxygen
